# Techno-economic evaluation of biogas production from food waste via anaerobic digestion

**DOI:** 10.1038/s41598-020-72897-5

**Published:** 2020-09-24

**Authors:** Abeer Al-Wahaibi, Ahmed I. Osman, Ala’a H. Al-Muhtaseb, Othman Alqaisi, Mahad Baawain, Samer Fawzy, David W. Rooney

**Affiliations:** 1 Department of Petroleum and Chemical Engineering, College of Engineering, Sultan Qaboos University, Muscat, Oman; 2 School of Chemistry and Chemical Engineering, Queen’s University Belfast, Belfast, BT9 5AG Northern Ireland UK; 3 Department of Animal and Veterinary Sciences, College of Agricultural and Marine Sciences, Sultan Qaboos University, Muscat, Oman; 4 International Maritime College Oman, Sohar, Muscat Oman

**Keywords:** Environmental sciences, Chemistry, Energy science and technology, Engineering

## Abstract

Food waste is a major constituent in municipal solid wastes and its accumulation or disposal of in landfills is problematic, causing environmental issues. Herein, a techno-economic study is carried out on the potential of biogas production from different types of food waste generated locally. The biogas production tests were at two-time sets; 24-h and 21-day intervals and results showed a good correlation between those two-time sets. Thus, we propose to use the 24-h time set to evaluate feedstock fermentation capacity that is intended for longer periods. Our approach could potentially be applied within industry as the 24-h test can give a good indication of the potential substrate gas production as a quick test that saves time, with minimal effort required. Furthermore, polynomial models were used to predict the production of total gas and methane during the fermentation periods, which showed good matching between the theoretical and practical values with a coefficient of determination R^2^ = 0.99. At day 21, the accumulative gas production value from mixed food waste samples was 1550 mL per 1 g of dry matter. An economic evaluation was conducted and showed that the case study breaks-even at $0.2944 per cubic metre. Any prices above this rate yield a positive net present value (NPV); at $0.39/m^3^ a discounted payback period of six years and a positive NPV of $3108 were calculated. If waste management fee savings are to be incorporated, the total savings would be higher, increasing annual cash flows and enhancing financial results. This economic evaluation serves as a preliminary guide to assess the economic feasibility based on the fluctuating value of methane when producing biogas from food waste via anaerobic digestion, thus could help biogas project developers investigate similar scale scenarios .

## Introduction

The high population growth rate and uncontrolled urbanization have created critical problems of solid waste disposal. A study performed by Baawain et al.^1^ confirmed that food waste is usually a major portion of any municipal solid wastes (MSWs) which are commonly disposed of in landfills or dumping sites, causing environmental problems. However, landfilling is expensive, requires space and can have a negative environmental impact if not well managed due to the production of leachate, methane and carbon dioxide and other nuisances like flies, odour, and vermin like birds and rodents. Leachate could also pollute underground water and soil along with the release of methane which is a potent greenhouse gas with a short-term global warming potential that is 84 times more powerful than carbon dioxide^2–4^. On the other hand, using food waste as a potential source for the production of sustainable fuels will complete the full cycle of this waste stream sustainably and thus, directly support and facilitate the concept of the circular economy in the form of open-loop recycling^5–8^. One of the promising ways of dealing with such waste stream is through processing via anaerobic digestion (AD) to produce biogas^9,10^. The AD is a series of processes in which microorganisms break down biodegradable material in the absence of oxygen for industrial or domestic purposes to manage waste and/or to release energy. Biogas is mainly composed of methane and carbon dioxide, with trace elements of gases such as hydrogen sulfides, ammonia and water vapour. There are several possible uses of biogas such as in cooking, heating, electricity generation, etc. The establishment of sustainable waste management practices that are effective, affordable, promote health and safety benefits to the public, prevent soil, air and water contamination, conserve natural resources, and provide renewable sources of energy that are generally environment friendly must be the priority.

The microbial population and type of microbes play a significant role in AD and affect the composition of biogas, which is produced due to four groups of microorganisms, fermentative, syntrophic, acetogenic and methanogenic bacteria^11–13^. These microorganisms normally occur in a natural environment and play different roles in the process of waste anaerobic degradation. Different microorganism types have different suitable environmental conditions to survive. The mesophilic bacteria is a type of organism that grows in a moderate temperature range of 20–45 °C with an optimum temperature of 35 °C^14^. On the other hand, thermophilic bacteria is a type of organism that optimally grows and survives in relatively hot temperatures (temperature range 41–122 °C), while the typical thermophilic condition is between 50 and 65 °C and 55 °C is optimum^15^. Microorganisms have a critical role in the degradation of organic substances, and it plays an important role in the anaerobic degradation process^16^. The volumetric amount of biogas produced in different digesters throughout the digestion time showed that mesophilic AD is more stable than thermophilic digestion^17^. Also, it required less process heat and hence less operating cost. However, thermophilic digestion allows a higher amount of feed loading with lower retention time, due to its higher conversion efficiency. The disadvantages of thermophilic AD are the degradation of enzymes and deficiency of the elements which are caused by the high temperature.

The efficiency of AD in biogas production is highly dependent on the process of biodegradation, where operating at an optimum condition increases the process efficiency^18^. There are important factors that influence biogas production such as temperature, hydraulic retention time, organic loading rate, inoculum, pre-treatment, feeding pattern and pH which play a major role in the AD process. Where pH influences the microorganisms’ growth through the process. Each bacteria type has a specific range of pH where it becomes active. For example, a suitable pH for methanogens bacteria is more than 6.5, however, the optimum pH value for acidogenic bacteria is in the range of 5–6.5. Sitorusa et al.^19^ performed a study on AD of mixed fruit and vegetable wastes to investigate the amount of biogas produced and variation of temperature and pH throughout the process. The experiments took place on 160 kg feedstock for 15 weeks. The fluctuation of temperature and pH during the process in the digester showed that the digestion process is running mostly at the mesophilic condition. There is a slight increase in the temperature of the digester during the first two weeks of the process from 28 up to 32 °C. After that, it increased sharply and reached the highest value in week 5 (~ 46 °C), this may have happened due to an ambient condition or due to the activity of the microbes. Then, for the following weeks, it dropped to the range of 32–37 °C, thus the temperature variation should be controlled so that it does not exceed a certain range. The reduction of pH in the AD process after the first three weeks can be explained mostly due to the formation of a high concentration of fatty acids in the digester and hence the accumulation of acid. At this stage, acidogenic bacteria start working and produce organic acid which leads to a decrease in the pH of the digester. After 9 weeks, the methanogens phase starts in the digester where the methanogens consume the acids. In general, controlling the pH inside the anaerobic reactor is not easy; hence, a basic solution may be required to be injected to maintain the pH within the optimum range (Sitorusa et al.^19^).

The substrate composition is crucial in the AD process, where the degradability of the feedstock varies and hence the optimum condition varies with the diversity of the composition in the feedstock^20,21^. Generally, the concentration of lipids, proteins and carbohydrates in the substrate gives a general idea about its behaviour in the AD process. Carbohydrates, due to its high degradability and rapid transformation, resulted in higher biogas yield^22^. However, although lipids give biogas with higher quality, they have a lower biodegradability rate, thus a longer residence time in the AD process is required. Li et al.^18^ studied twelve different types of food waste samples to investigate the effect of proteins, carbohydrates and lipids on AD and the amount of methane produced. Different food waste possesses different organic compositions which leads to a change in the methane yield. There was a remarkable difference between the peak pattern of methane production within samples, where 73 wt% of the carbohydrate composition showed the peak value of the methane yield within the first 11 h. At that peak, up to 98 wt% of the total methane was produced and this was explained since carbohydrates are a rapidly and easily degradable substance. On the other hand, the time taken to reach the peak methane production for the samples with higher protein and lipids and lower carbohydrates was in a range between 196 and 409 h, this is mainly due to a lower hydrolysis rate of proteins and lipids compared to carbohydrates. As an effective biological pre-treatment, rumen fluid was utilised which increased the biogas production by 66.5%, where the optimum time achieved was at 24 h^23^. This is in agreement with the work of Cattani et al.^24^. Furthermore, Kulivand and Kafilzadeh reported that 80% of gas production was achieved within 21 h using the rumen fluid^25^. Li et al.^26^ reported an optimum methane production of 14.9 mL after around 72 h, and Bachmann et al.^27^ reported that the optimum gas production for beans samples was at around 16 h. Dagaew et al.^28^ performed 96 h of biogas production in the AD process and found out that the optimum gas production was achieved at around 24 h.

The retention time in an anaerobic digester is specified based on the feedstock^29,30^. The biogas production varies throughout the digestion period^31^. This is mainly due to the variation of the pH which is a result of acid concentration increase/decrease. A digestion experiment ran for 10 days by Ziauddin and Rajesh^32^ on mixed kitchen waste to investigate the variation of biogas production from the first day to the last day of the experiment’s period. The biogas production sharply increased during the first 3 days from 80 to 120 mL from the first to the third day. After that, due to acid production and hence the increase in the acid concentration, pH was reduced in the process medium. As a result, biogas production declined to 50 mL and then increased but never reached the maximum value of 120 mL that was previously reached after 3 days of production. Apart from food waste, cow dung also contains biodegradable materials that can be converted to biofuel, yet gas yield from cow dung is low due to the low organic load and high nitrogen concentration^33^. However, food waste with its high nutrient content is a promising source for producing bioenergy^34,35^.

Herein, we investigated biogas production potential from food waste that is produced locally within an Omani oil company (Fahud cluster in Petroleum Development Oman) as a case study for the waste management. The study included various samples of food waste; two types of mixed food wastes (fruit and vegetable waste), bread waste, potato peels waste, meat waste, rice waste, dates fruit, legume beans, leafy vegetables and fish waste. Firstly, we measured the dry matter and nutrient composition in each food waste sample. Then we investigated the invitro true digestibility gas production to calculate the amount of gas produced from each sample. Furthermore, methane gas determination was carried out using DAISY incubator and gas chromatography. Finally, we performed a techno-economic study for biogas production derived from food waste provided in the case study. The utilisation of such waste stream in the production of sustainable biogas fuel will aid in the upcycling of problematic food waste by adding value and other techno-economic potential routes for application in the energy sector.

## Results and discussion

### Characterization results

Water content in food waste varies widely depending on the food source, and in some cases it reaches 75%^36^. Thus measuring the moisture content of each food waste sample is crucial when calculating the total quantities produced and the nutrient content in each sample^37^. Table 1 shows the dry matter content for the samples studied herein. The highest moisture content and hence the lowest dry matter present was the fruit and vegetable food waste sample, showing 80.4 and 19.6 wt%, respectively. The highest dry matter 81.7 wt% was observed in the date fruit sample, followed by bread waste sample with 77.2 wt% dry matter. The mixed food waste samples and potato peel sample contained a similar amount of dry matter of (± 25 wt%). Approximately 43 wt% of the meat waste sample was the dry matter, while for legume beans, rice, leafy vegetables, cow dung and fish waste (arranged in order from highest to lowest) were in the range between ~ 30 and 40 wt% dry matter.

**Table 1:** Proximate analysis for the samples along with their wt% of dry matter.

Samples	Crude fibre %	Ash %	Nitrogen %	Crude protein %	Fat %	Carbohydrate %	Dry matter %
Mixed food-1	1.45	2.18	1.97	12.33	1.13	10.41	27.5
Fruit and veg	1.62	1.72	0.42	2.65	0.30	13.33	19.6
Bread	0.65	1.03	1.85	11.30	9.07	55.15	77.2
Potato peels	1.49	1.62	1.62	10.13	2.81	10.05	26.1
Mixed food-2	1.62	1.35	0.92	5.78	2.07	14.68	25.5
Meat	0.23	1.14	6.04	37.74	0.51	3.48	43.1
Rice	0.28	0.74	1.64	10.27	0.23	26.28	37.8
Cow dung	6.50	5.44	2.40	15.00	0.53	4.53	32.0
Date fruits	2.08	1.43	0.36	2.26	0.16	75.77	81.7
Legume beans	2.49	1.63	1.86	11.62	0.59	23.07	39.4
Leafy veg	4.51	5.16	1.65	10.30	0.83	14.70	35.5
Fish waste	0.20	6.35	2.87	17.92	4.08	3.15	31.7

Carbohydrate % = 100 − (moisture content + crude fiber + ash + crude protein + fat) %.

To establish a relationship between biogas production performance and biochemical components, proximate analysis was performed for the samples studied herein as shown in Table 1. The analysis of the samples was performed on a dry basis and in duplicate for data reproducibility. The biogas formation rate increased with the increase in fibre content along with the decrease in fat content within the sample. Fats are considered as complex compounds that lead to an overall decrease in the biodegradation rate for the feedstock. For instance, the date fruits sample showed relatively higher fibre content along with the lowest fat content among the studied samples with values of 2.08 wt% and 0.16 wt%, respectively. Besides, the sample with the highest fat content had the lowest potential for biogas production, which was due to the complexity of the fat compound that required more residence time for degradation and biogas formation (i.e. fish waste). Furthermore, the sample with the highest protein content had a lower potential for biogas production. This is a result of the ammonia released during the degradation of protein, which caused an increase in pH and decreased the biodegradation rate by inhibiting the microorganisms in the AD system (i.e. meat sample). To sum up, for a better biogas production rate, a combination of various factors, such as high fibre content, high carbohydrate, low fats and protein contents, is required. Consequently, samples of date fruit, rice waste, legume beans and mixed food waste samples possessed a greater potential for high biogas production rate.

### Biogas production (24 h-time intervals)

**Figure 1:**
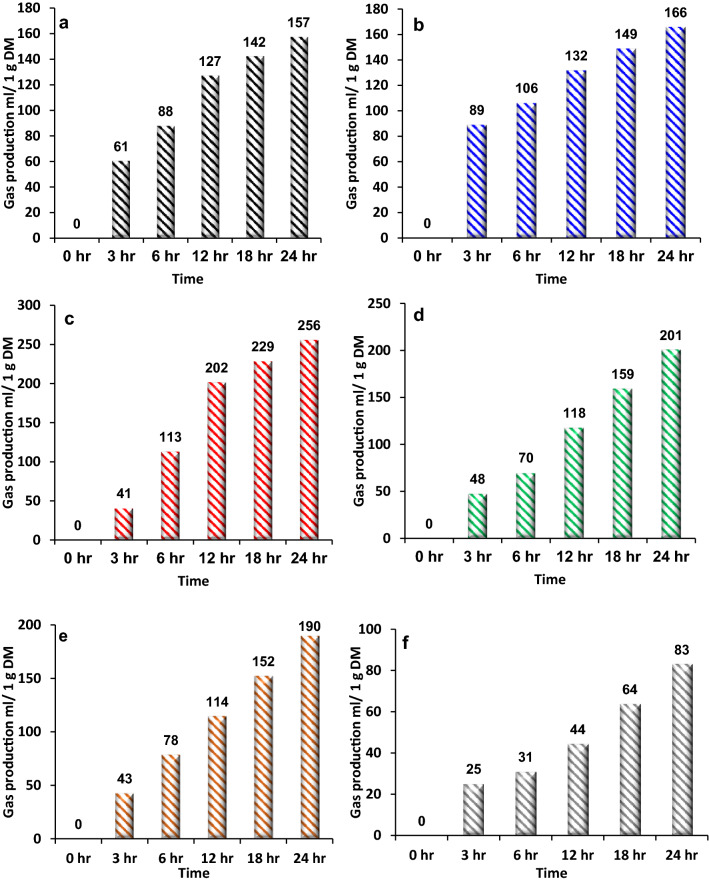
The gas production profile for (**a**) mixed food-1, (**b**) fruit and vegetables, (**c**) bread, (**d**) potato peel, (**e**) mixed food-2 and (**f**) meat samples over 24 h period.

**Figure 2:**
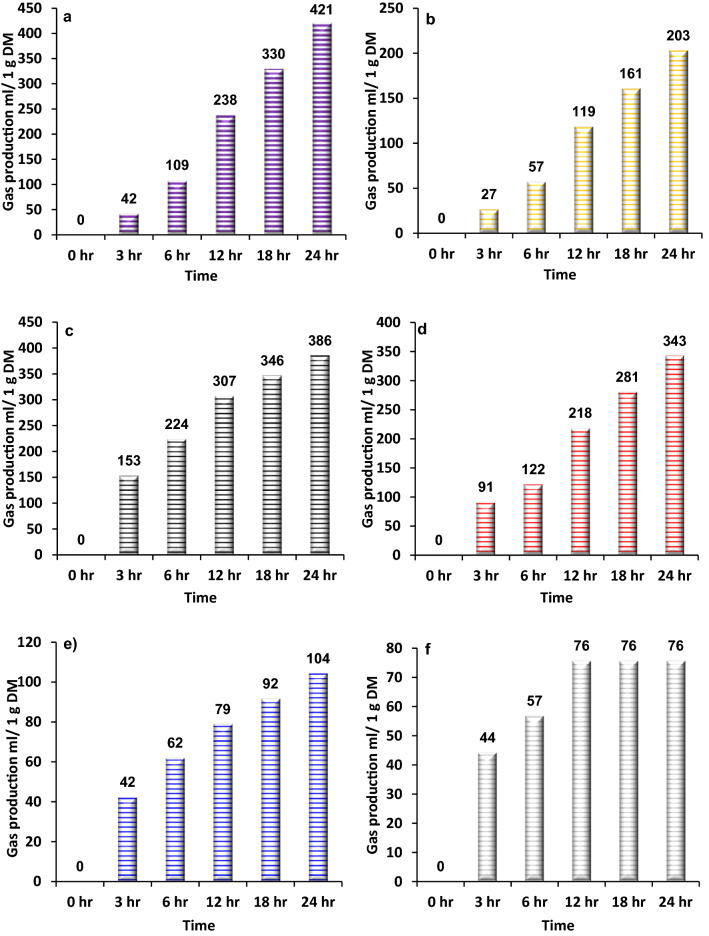
The gas production profiles for (**a**) rice, (**b**) cow dung, (**c**) date fruit, (**d**) legume beans, (**e**) leafy vegetables and (**f**) fish waste samples over 24 h period.

The total gas production from each sample was recorded in 3 h intervals for 24 h as shown in Figs. 1 and 2. The mixed food-1 sample showed a sharp increase in the gas produced during the first 3 h (61 mL/1 g DM), followed by a slight increase after 6 h (88 mL/1 g DM), then a sharp increase after 12 h (127 mL/1 g DM). Then up to 24 h, the increasing rate was almost stable, with total gas production of 157 mL/1 g DM. Similarly, the fruit and vegetable sample showed a sharp increase in the first 3 h up to 89 mL of gas per 1 g of dry matter of the sample. Then, from 3 to 24 h, the gas production rate slightly increased at a stable rate with maximum gas production of 166 mL of gas per 1 g of dry matter of the sample, similar to the conclusion obtained from Deressa et al.^38^. Regarding bread waste samples, as discussed earlier, a high dry matter content was noted and hence a high organic matter content. In general, bread waste contains a high amount of sugars, fibres and fats. Such organic-rich waste materials are a promising substrate to be used in the AD process with a high potential for biogas production. The bread sample gas production profile is shown in Fig. 1. The gas production rate slightly increased during the first 3 h (41 mL/1 g DM), then, sharply increased between 3 and 12 h to reach an approximate amount of 202 mL of gas per 1 g of dry matter. After 12 h, the gas production rate showed a slight increase up to 256 mL at 24 h.

Potato peel waste is rich in carbohydrates and hence is easily biodegradable^39^. Figure 1 shows a slight increase in the gas production profile at the first 6 h (70 mL/1 g DM), followed by a sharp increase in gas production up to 24 h with a maximum of 201 mL/1 g of dry matter. The results herein agree with the recent publication on potato peel waste that produced 217 mL/g^40^, where the physicochemical properties of potato peels indicated that it has a high potential for biogas production through AD^40^. The mixed food-2 sample showed almost a proportional relationship between retention time and gas production rate. The gas production volume steadily increased to reach 190 mL/1 g DM. Cattani et al.^24^ used similar technology and reported that food waste produced 168 mL/1 g DM of biogas. The slight difference in results may be due to the difference in the nutrients contained in each feedstock (i.e. different types of mixed food). Due to its high protein content, the meat waste can adversely affect the AD process by inhibiting the microbes through the production of ammonia which results in the digestion process. Figure 1 shows that the gas production rate slowly increased during the first 3 h (25 mL/1 g DM). Then, a slow production rate up to 24 h is noted with a maximum gas volume of 83 mL/1 g DM. It is not surprising that the meat sample showed poor gas production as it requires a long retention time, which could reach 80 days^41^, where during the first 10 days, slow biogas production is observed. This behaviour is due to the high protein content in the meat, which requires more time for degradation. Furthermore, carbohydrate content, which is the main source for energy supply for fermenting microbes, was the lowest in meat and fish samples, which may in part explain the low gas production in these two samples. Similar to protein meat content evaluated herein, Alqaisi et al.^42^ found a low gas yield for plant-rich protein sesame meal (i.e. crude protein content = 37.7%) compared to a significantly greater gas yield in rich carbohydrate feeds such as potato peels.

Figure 2 shows the gas production profile of the rice sample with a slight increase in the first 6 h (109 mL/1 g DM), followed by a sharp increase between 6 and 24 h, with total gas production of 421 mL per 1 g of dry matter of the sample. Therefore, herein the results showed that rice waste (cooked rice) could be considered as a promising source for biogas production. The above results are similar to Glivin and Sekhar^43^ results, where a comparison between the feasibility of biogas production between rice and vegetable waste showed that higher biogas was produced from rice waste compared to vegetable waste. That, as stated above, is due to the higher carbohydrate content within the rice waste. The gas production from cow dung shows a similar production to the mixed food sample, which is, in part, attributed to the similarity in organic matter content. Figure 2 shows that the gas production volume slightly increased and is stable in the first 6 h of the incubation process. Thereafter, gas production sharply increased in the period between 6 and 24 h (203 mL/1 g DM). Numerous studies were conducted on cow manure to investigate the biogas production rate. Putria et al.^44^ conducted an AD experiment on cow dung where 200 mL/g of biogas was produced in 24 h, which agrees with the results obtained in the current study. Ziauddin and Rajesh^32^ performed a study to compare the amount of biogas derived from food waste and cow dung. Two sets of samples were collected, set-1 contained cow dung and set-2 contained kitchen waste, where AD experiments were conducted on both samples for 8 days. The study revealed that food waste produced more gas than cow dung during eight days with average values of 89.37 and 23.75 mL, respectively. Another research was conducted by Chibueze et al.^45^ to evaluate the efficiency of biogas production from cow dung versus food waste and the same conclusion was obtained. The AD experiment was performed for 15 days on 150 g of two samples (cow dung and food waste feedstock). The results showed that after 15 days, 19.2 mL of biogas was produced from cow dung digestion, however, 30.58 mL was produced from food waste digestion. The result was predicted due to two factors: firstly, the nutrients contained in food waste are greater than those in cow dung. As per proximate analysis results, the cow dung contains less carbohydrate than that of food waste with values of 20 wt% and 61.9 wt%, respectively. Secondly, due to pH variation during the AD process in each sample. As pH is an important factor that affects digestion efficiency, pH values were measured throughout the process. It was observed that pH decreased more rapidly for the cow dung sample due to production of acids (ex. fatty and amino acids) which was a result of the high protein content, so became more acidic at the fourth day of the experiment. On the other hand, this took place on the 12th day for food waste sample (lower protein content). The acidity leads to depression in pH and hence reduces the efficiency of the anaerobic digestion. Thus, gas production volumes (mL) of food waste and cow dung after 15 days of continuous production were 30.58 and 19.2 mL, respectively.

The date fruits are cellulosic compounds which mainly contain sugars with a minor contribution from minerals and fats. Hence, it has considerable potential for biogas or biofuel production through the AD process, where date fruits produced double the amount of gas produced from cow dung. The gas production profile of the date fruits sample showed a sharp increase during the first 3 h (153 mL/1 g DM). Then, between 3 and 12 h, it increased to double the amount of gas (307 mL), then at 24 h, the total gas production was 386 mL per 1 g of dry matter of the sample.

Regarding legume beans, due to its high organic content, it can be used as a substrate for biogas production. Herein, a mixture of different types of legume beans (i.e. lentils, broad beans, chickpeas and beans) has been used. Figure 2 shows a sharp increase in the gas production rate at the beginning of the digestion process (between 0 and 3 h). Then, a slight increase was observed from 3 to 6 h. Beyond the 6 h retention time, the gas production rate sharply increased (from 122 mL at 6 h up to 343 mL at 24 h). Whereas for leafy vegetables, a mixture of several types of leafy vegetables which were commonly produced domestically (i.e. lettuce, coriander, mint and bay leaf) was used as feedstock for the AD experiment to explore their potential for biogas production^46^. The leafy vegetables gas production profile shows a slow increase in gas volume during the first 3 h (42 mL/1 g DM). Then, between 3 and 24 h, gas production increased almost proportionally and slightly with retention time, with a maximum amount of gas of 104 mL/1 g DM. Finally, the fish waste sample showed a slow increase in gas production at the beginning of the AD process (44 mL of gas produced up to 3 h). Then, gas production slightly increased to reach 76 mL after 12 h. After that, no change was observed in the amount of gas between 12 and 24 h. Kafle and Hun Kim^47^ performed an AD experiment on fish waste, but with a longer retention time (60 days). The results showed a similar trend of higher gas productivity at the beginning of anaerobic digestion, then a slowdown in biogas production rate.

**Figure 3:**
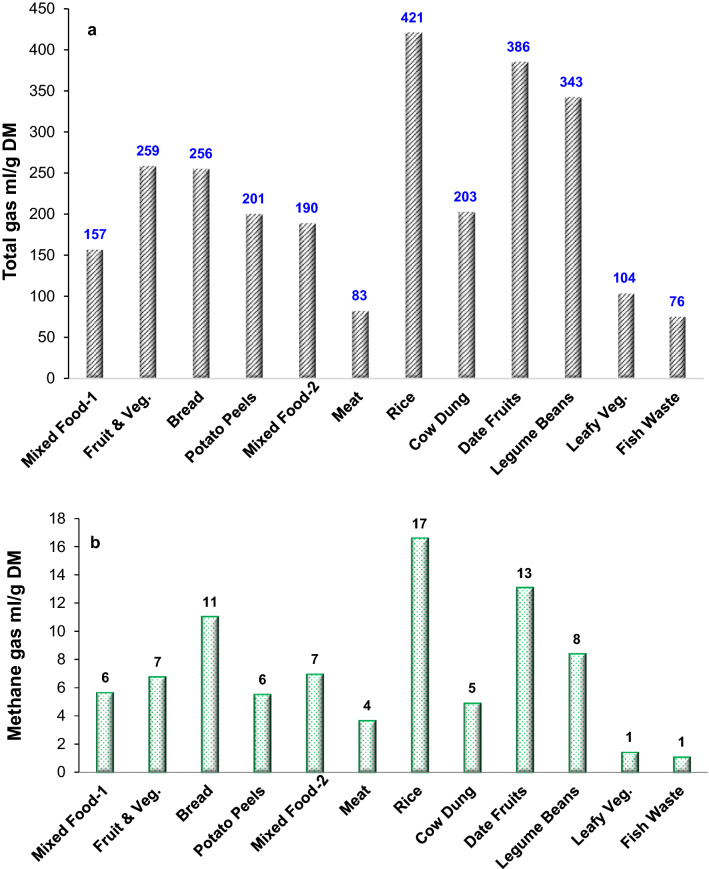
The total gas production profiles (**a**,**b**) the methane gas production over the waste samples studied herein, both for 24 h incubation period.

Interestingly, the samples with similar fat contents behaved similarly with regards to biogas production volume during the first 3 h. For instance, samples of fruits and vegetables and legume beans showed gas production of 89 and 91 mL/1 g DM, respectively. While samples of meat and cow dung with fat contents of 0.51 and 0.53 wt% showed gas production of 25 and 27 mL/1 g DM, respectively. The results herein agree with Li et al.^18^ study where the effect of carbohydrate, lipids and protein on AD was investigated. They reported that the feed with the highest carbohydrate content resulted in a higher biodegradation rate. On the other hand, the lipid content required more residence time to degrade because of its complex structure. Figure 3 shows the comparison between the samples studied herein in terms of the total gases along with the methane gas produced within the 24 h test. It is obvious that the highest three biogas production rates were for rice, date fruit and legume beans with total gas production of 421, 386 and 343 mL/1 g DM, respectively as seen in Fig. 3a. Methane gas production for those samples showed 17, 13 and 8 mL/1 g DM, respectively as shown in Fig. 3b. Based on their composition that favours biogas production and local availability, we decided to run biogas production tests for a longer period (21 days) for those samples (rice, date fruit and legume beans) along with mixed food waste as an abundant waste material in this case study.

### Biogas production and methane concentration (21 days-time intervals)

**Figure 4:**
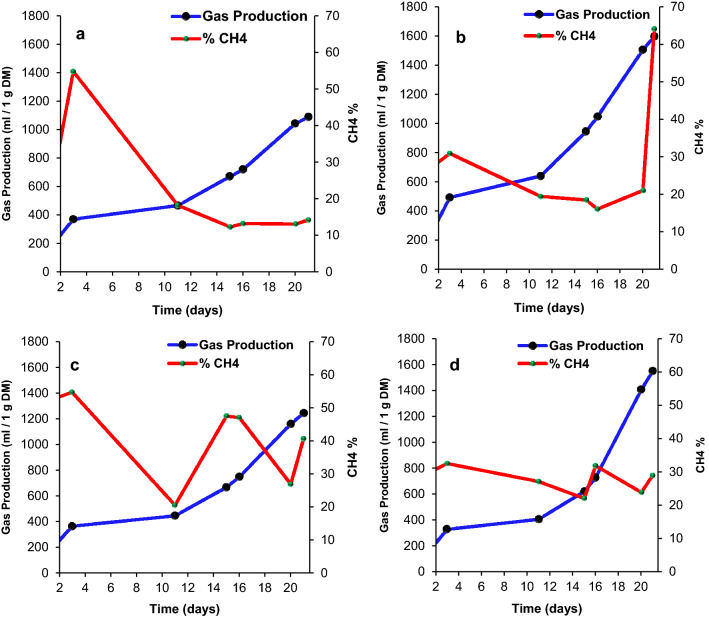
The total gas production profiles and % methane gas production over 21 days using (**a**) date fruit, (**b**) rice waste, (**c**) legume beans and (**d**) mixed food waste samples.

The biogas production along with methane concentration over 21 days is shown in Fig. 4a–d for the selected samples (the date fruit, rice waste, legume beans and the mixed food waste). Overall, in each sample, there was a daily increase in the accumulated produced biogas. At day 21, the highest gas production values from the rice waste and mixed food waste samples were of ~ 1600 and 1550 mL/1 g DM, respectively. Figure 4a–d show fluctuation in the methane concentration in all samples. The fluctuation in methane concentration was attributed to the large fluctuation in the levels of methanogenic population bacteria, as volatile fatty acids were accumulated and then subsequently consumed. Similar performance (i.e. fluctuation in methane concentration) was reported by Griffin et al.^48^. The study focused on analysing the performance of a mesophilic anaerobic digester where they observed a variation in the amount of produced biogas and methane concentration throughout the digestion process. Different digestion processes took place in the incubation period which resulted in a change in the pH of the incubator environment. The microorganisms’ growth through the AD process is influenced by the variation in the pH value. Each bacteria type (i.e. methanogenic) has a specific range of pH to be active and that is reflected by the fluctuation in methane concentration in each sample. Furthermore, the temperature fluctuation in the digester affects the type/population of the microorganisms. Normally, there were two sources of energy (temperature change) inside the incubator, the surrounding condition and the microorganism’s activity. On the other hand, the nutrients content (i.e. composition) of the feedstock played a vital role in the amount/dynamics of biogas produced as shown in Table 1.

In the date fruit sample, the methane concentration was less than 20% in day 1, and then it significantly increased to 55% in day 3. Thereafter, the methane concentration was reduced to less than 20% and remained at that low value until day 21. However, the rice waste sample showed a gradual decrease in the methane concentration from day 3 up to day 16, then it increased dramatically to reach ~ 64% on day 21. The legume beans sample started with a high methane concentration (52%) on day 1 from the AD and then fluctuated to reach 40% by day 21. The methane concentration of the mixed food waste sample fluctuated at around 30% throughout the AD process.

### Modelling results of biogas production using food waste

Table S1 (Supplementary Information) shows the total gas and methane production of different waste samples. There was a significant variation (p < 0.05) in gas and methane production between wastes. Gas production varied between 76 and 421 mL/g DM in fish waste and rice waste samples, respectively. Moreover, methane production varied between low production level of 1 mL/g DM and high production of 16.6 mL/g DM in fish waste and rice waste samples, respectively. The 24 h gas and methane production evaluations could be interesting to map potential gas production in waste samples, as it provides preliminary results on fermentation potential. In our study, there was a strong correlation (corr = 0.92) between the in vitro gas and methane production at 24 h. The considerable variation in gas and methane production could be partly explained by the variations in nutrient contents of wastes. Such variations provide nutrient supply to the microbes thereby resulting in different gas production levels. The evaluated waste samples herein represent a wide range of industrial and farming wastes, therefore variations in their nutrient contents are expected. Overall, the 24 h test might be used to evaluate feedstock fermentation capacity that is intended for longer periods. Thus, our approach could have a potential application in industry as the 24 h test can give a good indication of the potential substrate gas production as a quick test that saves time, with minimal effort required.

**Figure 5:**
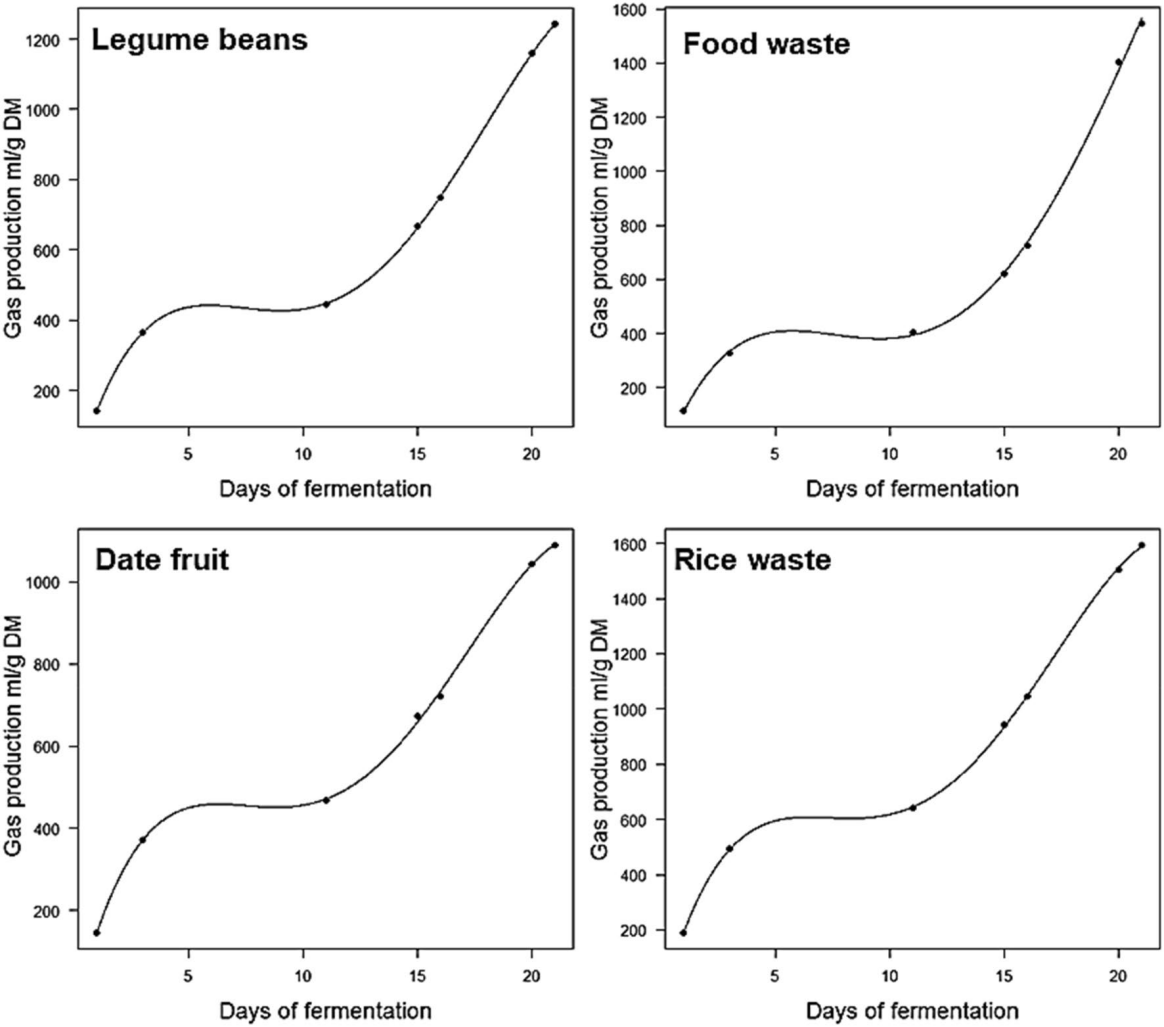
Polynomial graphs of gas production (mL/g DM) concerning fermentation time in four food waste samples. Lines represent the predicted gas and dots represent practical values.

**Figure 6:**
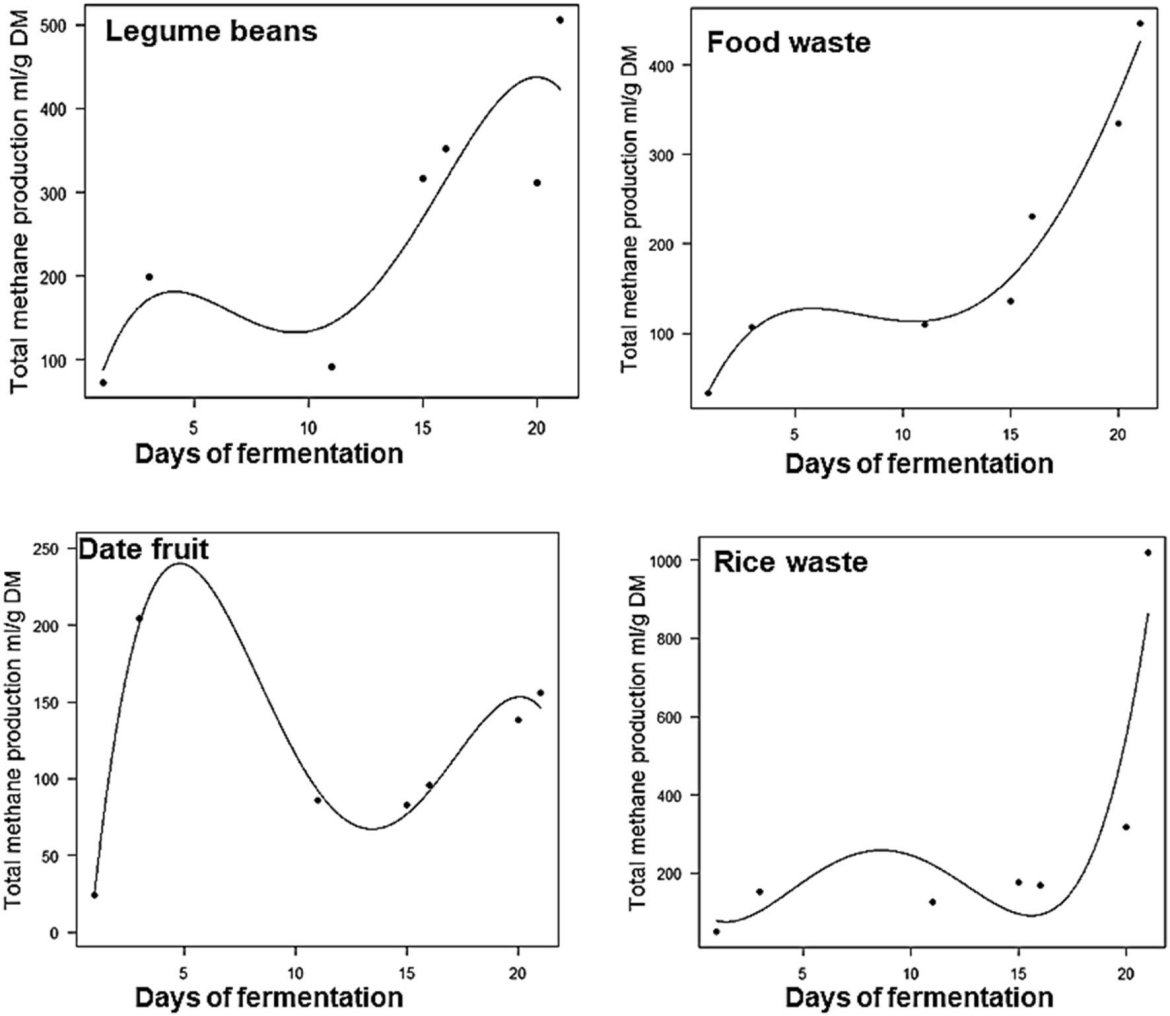
Polynomial graphs of methane production (mL/g DM) concerning fermentation time in four food waste samples. Lines represent the predicted gas and dots represent practical values.

Table 2 presents a summary of the polynomial models used to predict the production of total gas and methane during the fermentation periods. Recent studies have suggested that polynomial models are particularly suited for examining gas production over the fermentation period in different fermentation systems^23,49–51^. From a fermentation standpoint, gas production is a continuous process and might show a growth relationship over a period of time in which such relationship can be tested by a polynomial model. The gas production models (Figs. 5, 6) could explain the majority of data points, this conclusion is supported by the high goodness of fit. The total gas models showed an excellent fit between the theoretical and practical data. It is not surprising that the methane model did not show a good matching between the theoretical along with the practical data, except for only two samples, the date fruit and food waste samples. This is maybe due to the fluctuations observed in the methane production as shown in Fig. 4. Methane production is proportional to gas production, this proportion changes over fermentation days depending on the nutrient contents of the biowastes. Therefore, in our study, the polynomial model used might only be a good fit for methane production data in two samples (date fruit and food waste). Further parameters such as nutrient contents and the interaction between total gas production and methane production level can be introduced in future studies to improve the fit.

**Table 2:** Summary polynomial models for gas and methane production from date fruit, rice waste, legume beans, and food waste samples.

	Model	Adjusted R^2^	P-value
**Biogas production models**			
Date fruit	Y = − 50.9 + 230X − 36.6X^2^ + 2.3X^3^ − 0.04X^4^	0.99	< 0.0001
Rice waste	Y = − 72 + 308x − 49x^2^ + 3.19X^3^ − 0.06X^4^	0.99	< 0.0001
Legume beans	Y = − 56.29 + 232.9x − 37x^2^ + 2.3x^3^ 0.04x^4^	0.99	< 0.0001
Food waste	Y = − 86 + 229.5x − 36x^2^ + 2.1x^3^ − 0.03x^4^	0.99	< 0.0001
**Methane production models**			
Date fruit	Y = − 135.8 + 191x − 31x^2^ + 1.8x^3^ − 0.036x^4^	0.94	0.042
Rice waste	Y = 110–54.5x + 23x^2^ − 2.4x^3^ + 0.07x^4^	0.55	0.29
Legume beans	Y = − 3.9 + 112.9x − 22.6x^2^ + 1.63x^3^ − 0.04x^4^	0.36	0.37
Food waste	Y = − 22.7 + 67x − 9.9x^2^ − 0.54x^3^ − 0.008x^4^	0.91	0.059

### The economic evaluation of biogas production from food waste

A preliminary economic study was performed herein to evaluate the economic feasibility of installing an anaerobic digester unit to process food waste produced from the case study (Fahud camp). The main concept is the replacement of LPG with biogas, where savings can be achieved. Bhatt and Tao studied the economic perspectives of biogas production via AD at different facility scales^36^. It is important to note that if food waste is processed at higher plant scales this ultimately reduces the overall cost through economies of scale. This can be achieved through the development of a centralized food waste collection and processing approach. The estimated biogas production rate used in this economic evaluation is based on the value determined from the AD experiment, as shown below in Table 3 and Table S2 at a scale of 3280 kg/month of food waste.

**Table 3:** Estimated biogas production rate calculation in the case study.

1	Amount of food waste at Fahud—Fresh Matter @ 26.5% DM	3280 kg/month
2	Amount of food waste at Fahud—Dry Matter	869.2 kg/month
3	Amount of biogas produced derived from experiments	1550 L/kg
4	Methane content in biogas derived from experiments	30%
5	Amount of methane produced (mL/g). Calculation: [3] × [4]	465 mL/g
6	Total amount of methane based on amount of food waste Calculation: ([2] × [5] × (10^3^))/(10^6^)	404 m^3^/month

The economic analysis is based on the discounted cash flow (DCF) approach whereby projected future cash flows are discounted at a rate that represents the cost of capital. The analysis includes identifying the discounted payback period as well as the net present value (NPV) of the case study. The analysis investigated the impact of various gas prices on the viability of the project to provide investment guidance to decision-makers. Table 4 presents the assumptions on which the analysis is built.

**Table 4:** Assumptions for economic analysis based on the case study.

7	Gas value based on methane content (IRENA, reference)	$0.22–$0.39/m^3^
8	CAPEX (please refer to supplementary tables for a breakdown of the equipment, CAPEX based on a quotation from a Chinese manufacturer)	$6448
9	OPEX Annual (5% of CAPEX to cover maintenance, electric consumption and consumables). (Assumption provided by equipment manufacturer)	$322.4
10	Investment in Working Capital. Calculation: 3 years × [9]	$967.2
11	Total investment. Calculation: [8] + [10]	$7415.2
12	Project lifetime (provided by equipment manufacturer)	10 years
13	Discount rate/cost of capital (given)	8%
14	Tax—the project is tax-exempt (given)	0%
15	Depreciation is not accounted for since the project in the case study is tax-exempt. Depreciation will only have an impact on cash flow as a tax shield	Not accounted for
16	Gas price inflation (not accounted for to present a very conservative scenario)	Not accounted for

Table 5 presents the annual cashflow calculations on which the DCF model is built.

**Table 5:** Annual cashflow calculation for the economic analysis based on the case study.

17	Gas value based on methane content	$0.22	$0.26	$0.30	$0.34	$0.39
18	Annual income/savings calculation: [17] × [6] × 12 months	$1066.56	$1260.48	$1454.4	$1648.32	$1890.72
19	Annual cashflow calculation: [18] −[9]	$744.16	$938.08	$1132	$1325.92	$1568.32

Discounted Payback Period, DPP (years) is calculated according to Eq. (1)

1 $$DPP=\frac{\mathrm{ln}\left(\frac{1}{1-(I*r/CF)}\right)}{\mathrm{ln}(1+r)}$$

where I = initial investment, r = discount rate and CF = cash flow.

Where the net present value (NPV) is calculated according to Eq. (2)^52^.

2 $$NPV=-I+\sum_{t=0}^{N}\frac{{CF}_{t}}{{(1+r)}^{t}}$$

### Economic analysis results

Herein, Table 6 presents the discounted payback period and net present value (NPV) of the case study under different methane prices per m^3^, for a project lifetime over 10 years. Both analyses methods take into account the time value of money. The calculated net present value is the sum of all discounted future cash flows less the initial total investment made in capital expenditure and working capital. A positive NPV means that building a biogas unit is to be considered and that value is being created. If the NPV result is negative then the project in the case study should be discarded and if the result is zero, then no value is being created but also no loss is incurred. The calculated value is the total financial contribution of the case study over its lifetime to its owners, taking into account the time value of money. It can be noted that gas prices of $0.22/m^3^ and $0.26/m^3^ yield a negative NPV, indicating the project in the case study will be incurring losses at such rates. Furthermore, the discounted payback period calculated at these rates is > 10 years, i.e. longer than the expected lifetime of the equipment. It is not advised to carry out the project at such prices/m^3^. Furthermore, all the other rates investigated indicate that value is being created by developing this project since they all carry a positive NPV, however, payback periods vary accordingly. Furthermore, for the project to break-even, i.e. to yield an NPV of 0, a gas rate of approximately $0.2944/m^3^ is required. Any prices under this rate would yield losses, and any prices above this rate would create value. The investment in a Fahud biogas production plant should be carefully considered based on the current and anticipated future gas rates. Please note that the annual cash flows used in the analysis are based on gas savings only. If waste management fee savings are incorporated, the total savings would be higher, increasing annual cashflows and enhancing project results.

**Table 6:** Summary of discounted payback period along with net present value based on the different pricing of methane per m^3^.

Methane price/m^3^	$0.22	$0.26	$0.30	$0.34	$0.39
Discounted payback period	> 10 years	> 10 years	9.65 years	7.71 years	6.18 years
NPV	− 2421.83	− 1120.61	180.61	1481.83	3108.35

Furthermore, this amount of biogas can replace approximately 28.6% of LPG gas currently consumed in Fahud (for cooking purposes), as shown in Table 7.

**Table 7:** Summary for the LPG replacement for cooking purposes based on the case study.

20	Amount of LPG in once cylinder (given)	44 kg/cylinder
21	Density of LPG	0.505 kg/L
22	Volume of LPG in one cylinder (L/cylinder). Calculation: [20]/[21]	87.13 L/cylinder
23	Volume of LPG in one cylinder (m^3^/cylinder). Calculation: [22]/1000	0.0871 m^3^/cylinder
24	LPG expansion rate (given)	270
25	Amount of gas after expansion. Calculation: [23] × [24]	23.52 m^3^/cylinder
26	Number of cylinders that can be replaced by biogas per month. Calculation [6]/[25]	17.18 cylinders/month
27	Fahud LPG consumption per month (given)	60 cylinders/month
28	Reduction of consumption. Calculation: ([26]/[27]) × 100	28.6%

There are also drawbacks associated with biogas production which include the release of greenhouse gas emissions (GHG) due to the ineffective handling within the process.^53^. A life cycle assessment should be carried out to evaluate and ensure the environmental sustainability of such process^54^. It is worth noting that food waste could be used for the production of value-added chemicals such as carboxylic acids (C_2_–C_6_ acids) that may provide a better value proposition^5,55,56^.

## Conclusion

In conclusion, herein, we used different types of food wastes to produce biogas and carried out a techno-economic evaluation to investigate the financial viability of setting up a small scale biogas plant. Our results show varying gas production rates between 76 and 421 mL/1 g DM. The feed composition is responsible for the variations in biogas yields with wastes that are rich in carbohydrate and fibre content, such as rice waste, carry remarkable potential for biogas and methane gas yields. Furthermore, during the 21 incubation days of anaerobic digestion, a fluctuation in methane concentration was observed in all samples which ranged between 20 and 60%. A good matching was observed between the theoretical and practical data based on the polynomial models with R^2^ = 0.99. The economic evaluation results indicated that the project breaks-even at approximately $0.2944/m^3^, any prices above this rate yield a positive NPV. If waste management fee savings are incorporated, the total savings would be higher, increasing annual cash flows and enhancing project results. This economic evaluation serves as a preliminary guide to assess case study feasibility based on the fluctuating value of methane.

## Experimental

### Materials

Food waste samples were collected from Fahud Camp in PDO in Oman. The anaerobic digestion experiment was conducted on 11 different types of food waste in addition to a cow dung sample, to evaluate the biogas production performance from each sample. The following food waste samples were used: two types of mixed food wastes (fruit and vegetable waste), bread waste, potato peels waste, meat waste, rice waste, dates fruit, legume beans, leafy vegetables and fish waste.

### Characterisation techniques

#### Dry matter determination

The samples were weighed at ambient conditions and then placed in the oven and left at ~ 105 °C for 24 h. Then the dried samples were transferred into a desiccator. When the samples were cooled, the sample weight was recorded. Then the dry matter was calculated according to Eq. (3)

3 $$\%DM=\frac{Dry weight }{Sample fresh weight }\times 100 \%$$

After the drying procedure was completely done, 100 g of each sample is weighed in plastic containers and are labelled according to the dates of sample collection. The samples were ground in a coffee grinder until it was a fine powder.

#### Crude fibre (CF) content determination

1 g of dry sample was weighed into a crucible and the CF was measured with the aid of Fiber Tec system using100 mL of 0.128 M Sulphuric acid (first reagent). A few drops of octanol were added to prevent foaming, then the sample was heated to boiling by turning the effect control to max. The heat and boil were adjusted for 30 min by turning the timer. After 30 min the sample was filtered by turning the valve to vacuum position. The sample was washed three times with hot water. Then, 100 mL of sodium hydroxide solution (second reagent) was added to each sample. A few drops of octanol were added and boiled, as above, for another 30 min. After 30 min the samples were filtered and washed, as above, 3 times with hot water. The samples were washed 3 times with acetone and vacuum dried at 100 °C overnight. Then the samples were calcined in a muffle furnace at 500 °C overnight, then allowed to cool down and weighed. The percentage (wt%) of fibre in the test sample is given by Eq. (4):

4 $$CF=\frac{Dry weight-Ash weight}{Weight of sample}\times 100 \%$$

#### Ash determination

A specific weight of sample was added into a porcelain crucible, then calcined at 550 °C for 6 h. Then, the sample was cooled, and the crucible was weighed. The ash content was calculated according to Eq. (5)

5 $$Ash=\frac{Ash weight }{Fresh weight}\times 100 \%$$

#### Fat content determination

The samples were ground into the size of < 1 mm, then 1 g of sample (in duplicate) was weighed into an extraction thimble, plugged lightly with cotton wool and placed in the extractor. Followed by,100 mL of petroleum spirit added into a distillation flask and placed on the heaters. The solvent was heated at 50 °C and extracted for 8 h. After the extraction was complete, the heaters were stopped, and the flask was allowed to cool. Thimbles were removed from the soxhlet with the help of a pair of long forceps and placed in a beaker under the fume hood. Most of the solvent was distilled from the flask into the extractor. The flask (which now contains the fat) was de-attached and dried in an oven at 100 °C for 2 h to evaporate the remaining solvent. The flasks were cooled in a desiccator (for 45 min). The flasks were weighed again with the extracted oil and the fat content was calculated as in Eq. (6):

6 $$\mathrm{Fat}=\frac{(Flask weight after extraction-Flask weight before extraction)}{Sample weight}\times 100 \%$$

#### Determination of crude protein and nitrogen

Firstly, the digestion step was performed, 0.5 g (in duplicate) of the sample was weighed, then 10 mL of sulphuric acid was added. The tube was then placed in a digestion rack and digested for 1 h. After digestion, the tubes were allowed to cool. Then, this was followed by the distillation and titration steps and then calculated as per Eqs. (7) and (8)

7 $$\% {\text{ Nitrogen}} = {1}.{4} \times {\text{Molarity of the acid}}$$

8 $$\% {\text{ Crude Protein}} = {\text{Nitrogen }}\% \times {6}.{25}$$

#### Rumen liquor collection

**Figure 7:**
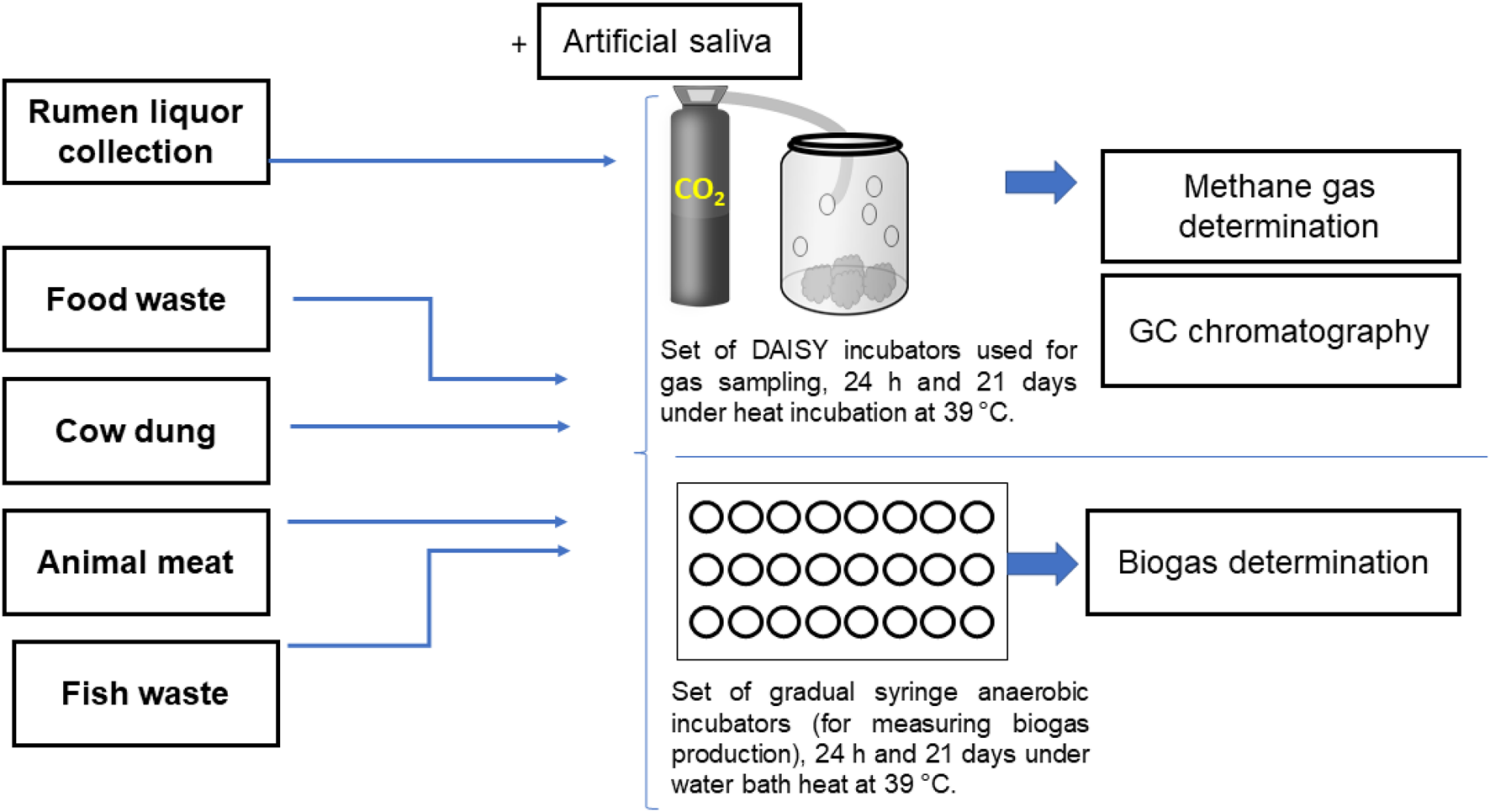
The illustration of the anaerobic experimental digester using different types of food waste samples.

Rumen liquor used in the experiments was collected from a fistulated cow in the “Agricultural Experiment Station, Sultan Qaboos University”. Water containers (preheated to 39 °C) were used to place the collected rumen fluid. The collecting devices prepared, funnel and filter were used. At all times the rumen liquor sample was covered and flushed with CO_2_ at 39 °C water bath. The illustration of the anaerobic experimental digester is shown in Fig. 7.

#### Invitro true digestibility manual gas production

The invitro digestible organic matters were determined in triplicate samples at lab-scale. 100 mL calibrated glass syringes were used to measure gas produced by fermentation. The syringes contained 200 mg of feed and were kept for 21 days. After mixing the fresh-made reducing solution the artificial saliva changed colour from navy blue to pink and finally, it became colourless. Approximately 30 mL of rumen liquor and artificial saliva mixture (with a ratio of 1–2) were added to the syringes, the plunger was adjusted. Finally, the syringes were kept in a water bath at 39 °C for the desired amount of time. Readings of the calibrated syringes were taken every 0, 3, 6, 12, 18, 24 h and then daily for 21 days.

#### Invitro true digestibility and methane gas determination

For ruminal methane determination, the daisy incubation method was used. The ground feed samples were weighed to 0.250 g and filled in nylon filter bags, which were previously rinsed with acetone to remove any traces of surfactant that might inhibit microbial digestion and dried in 100 °C in an oven. The collected rumen liquor was filtered through two layers of muslin cloth under continuous flushing of carbon dioxide gas to maintain the anaerobic condition and the buffer solution was prepared and kept in the one-way valve ankom daisy incubation jars for 24 h in vitro at 39 °C.

#### Determination of methane gas

Methane concentration in the gas produced was measured using the gas chromatography (GC) technique. Daisy jars were transferred from the incubator and brought to the GC, the rubber plug has a needle inserted in the headspace of the jar and injected in the GC. GC–MS analysis was performed on a Perkin Elmer Clarus 600 GC System, fitted with an Rtx-5MS capillary column (30 m × 0.25 mm i.d. × 0.25 μm film thickness; maximum temperature, 350 ºC), coupled to a Perkin Elmer Clarus 600 C MS. Ultra-high purity helium (99.99%) was used as a carrier gas at a constant flow of 1.0 mL/min. The injection, transfer line and ion source temperatures were 280, 290 and 290 °C, respectively. The ionizing energy was 70 eV. Electron multiplier (EM) voltage was obtained from autotune. All data were obtained by collecting the full-scan mass spectra within the scan range 40–550 amu. The injected sample volume was 1 μL with a split ratio of 10:1. The oven temperature program was 60 °C (held for 1 min) with a heating rate of 80 °C /min up to 280 °C, then held for 25 min. The unknown compounds were identified by comparing the spectra obtained with mass spectrum libraries (NIST 2011 v.2.3 and Wiley, 9th edition).

#### Modelling methods

Data on methane and biogas production at 24 h were analysed using the linear model (lm) procedure of R (R Core Team 2018). The Wald Chi-Squared test (Type II) was performed to obtain the least-square means of the tested variables.

The model fitted to the 24 h gas and methane data was as follows:

$$response variable=fixed effects+error$$

where the response variable was the gas and methane production in 24 h (mL/g DM), differences in results were considered significant if *p value* < 0.05.

A polynomial model was used to fit the 21 days biogas and methane production data. The quartic model used to predict biogas and methane production was as follows: Y = b_0_ + b_1_x + b_2_x^2^ + b_3_x^3^ + b_4_x^4^, model parameters were Y the response variable of total gas and methane gas production (mL/g DM), b_0_ is the intercept and b_1_ to _4_ are the model coefficients.

## Supplementary information

Supplementary file1
