# Supplementary material for: Techno-economic evaluation of biogas production from food waste via anaerobic digestion
Source: Sci Rep. 2020 Sep 24;10:15719. doi: 10.1038/s41598-020-72897-5 (PMC7515879; doi:10.1038/s41598-020-72897-5)

***Supplementary Information for***

**Techno-economic Evaluation of Biogas Production from Food Waste via Anaerobic Digestion**

Abeer Al-Wahaibi ^a^, Ahmed I. Osman ^b*^, Ala’a H. Al-Muhtaseb ^a*^, Othman Alqaisi ^c^, Mahad Baawain ^d^, Samer Fawzy ^b^, David W. Rooney ^b^

^a^ Department of Petroleum and Chemical Engineering, College of Engineering, Sultan Qaboos University, Muscat, Oman

^b^ School of Chemistry and Chemical Engineering, Queen’s University Belfast, Belfast BT9 5AG, Northern Ireland, UK.

^c^ Department of Animal and Veterinary Sciences, College of Agricultural & Marine Sciences, Sultan Qaboos University, Muscat, Oman

^d^ International Maritime College Oman, Sohar-Muscat

Corresponding Authors: Ahmed Osman, Ala’a H. Al-Muhtaseb

Email: [aosmanahmed01@qub.ac.uk](mailto:aosmanahmed01@qub.ac.uk), [muhtaseb@squ.edu.om](mailto:muhtaseb@squ.edu.om)

**Table S1:** Gas and methane production (ml/g DM) for different waste samples at 24 h

| **Waste type** | **24 h Gas ml/g DM** | **24 h Methane ml/g DM** |
| --- | --- | --- |
| Fish Waste | 75.8 | 1.0 |
| Meat | 83.2 | 3.8 |
| Leafy Vegetables | 104.4 | 1.6 |
| Fruit & Vegetables | 166.2 | 6.5 |
| Mixed food1 | 189.2 | 7.0 |
| Mixed food2 | 189.7 | 7.0 |
| Potato Peels | 200.5 | 5.3 |
| Cow Dung | 203.2 | 5.0 |
| Bread | 256.0 | 11.2 |
| Legume Beans | 342.8 | 8.4 |
| Date Fruits | 385.4 | 13.0 |
| Rice | 421.4 | 16.6 |
| SE | 22.9 | 0.65 |
| P value | < 0.0001 | < 0.0001 |

24 h gas results presented in the least-square means, differences in results were considered significant if p-value <0.05.

**Table S2:** Equipment list for the biogas production from anaerobic digestion.


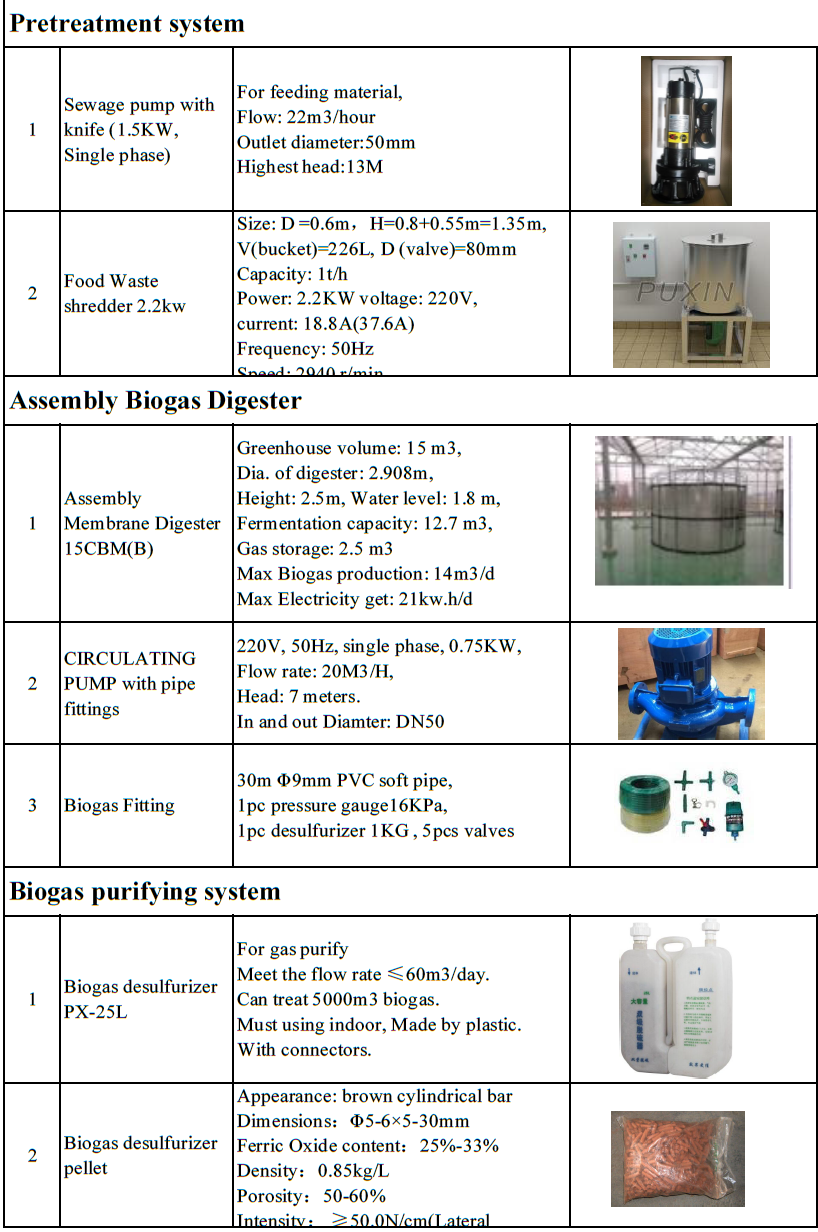


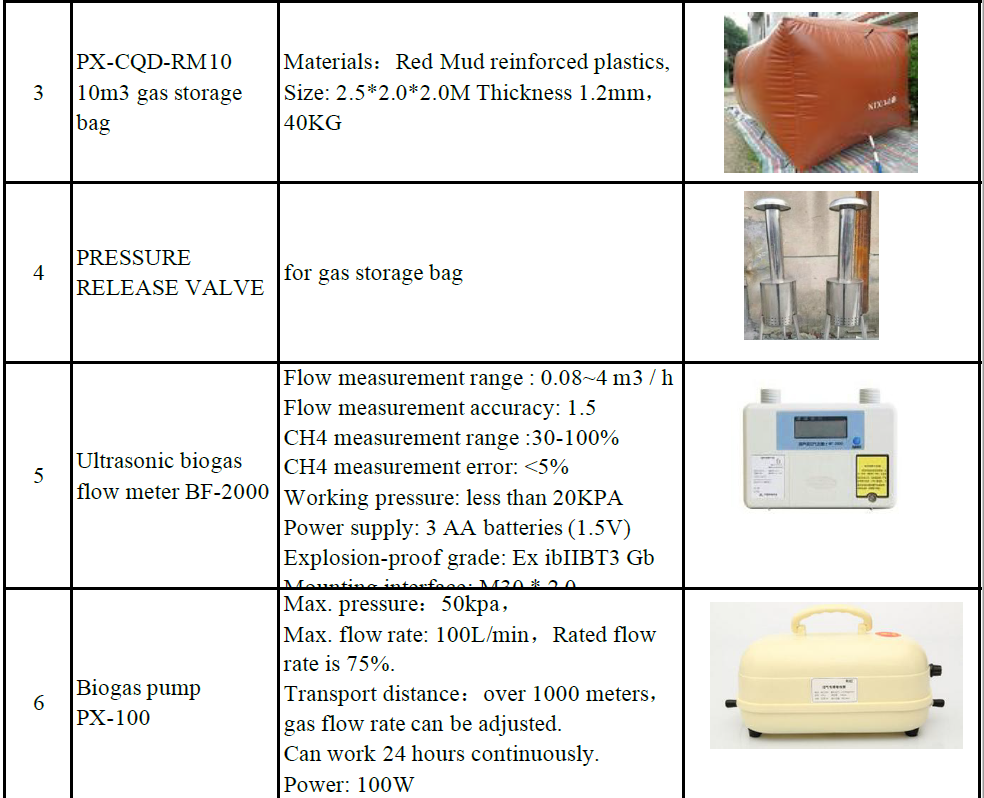

Supplement: Supplementary file 1 — Supplementary file1 [file 41598_2020_72897_MOESM1_ESM.docx]
